# Evaluation of the efficacy of pulsed electromagnetic field in the management of patients with diabetic polyneuropathy

**DOI:** 10.4103/0973-3930.53121

**Published:** 2009

**Authors:** Vinay Graak, Sarika Chaudhary, B. S. Bal, J. S. Sandhu

**Affiliations:** Department of Sports Medicine and Physiotherapy, Guru Nanak Dev University, Amritsar, Punjab, India; 1 Department of Medicine, Govt. Medical College, Amritsar, Punjab, India

**Keywords:** Diabetic polyneuropathy, nerve conduction velocity, pulsed electromagnetic field

## Abstract

**AIM:**

The study was carried out to evaluate and compare the effect of low power, low frequency pulsed electromagnetic field (PEMF) of 600 and 800 Hz, respectively, in management of patients with diabetic polyneuropathy.

**SETTINGS AND DESIGNS:**

The study was a randomized controlled trial performed in Guru Nanak Dev University and Medical College, Amritsar, India with different subject experimental design.

**MATERIALS AND METHODS:**

Thirty subjects within an age group of 40–68 years with diabetic polyneuropathy stages N1a, N1b, N2a were randomly allocated to groups 1, 2, 3 with 10 subjects in each. Group 1 and 2 were treated with low power 600 and 800-Hz PEMF for 30 min for 12 consecutive days. Group 3 served as control on usual medical treatment of diabetic polyneuropathy (DPN). The subjects with neuropathy due to any cause other than diabetes were excluded. The pain and motor nerve conduction parameters (distal latency, amplitude, nerve conduction velocity) were assessed before and after treatment.

**STATISTICAL ANALYSIS:**

Related t-test and unrelated t-test were used for data analysis.

**RESULTS:**

Significant reduction in pain and statistically significant (*P*<0.05) improvement in distal latency and nerve conduction velocity were seen in experimental group 1 and 2.

**CONCLUSIONS:**

Low-frequency PEMF can be used as an adjunct in reducing neuropathic pain as well as for retarding the progression of neuropathy in a short span of time.

## Introduction

Diabetic neuropathy is a common microvascular complication of diabetes over time and one of the major cause of nontraumatic amputations. A widely-accepted definition of diabetic peripheral neuropathy is “the presence of symptoms and / or signs of peripheral nerve dysfunction in people with diabetes after exclusion of other causes”.[1,2] Depending on criteria, DPN is estimated to occur in 50–90% of individuals with diabetes for more than 10 years.[3] The impairment of peripheral nerve function in diabetic individuals should be regarded not as a neurological complication but as a neurological manifestation of the disease.[4,5] It approaches 50% in most diabetic population, mainly with painful symptoms.[1] It may present as symmetric polyneuropathies, focal and multifocal neuropathies and mixed form of neuropathy. Distal symmetric sensorimotor polyneuropathy is the most common type of diabetic neuropathy and is characterized by the progressive loss of sensation and less frequently, motor function in a distal to proximal gradient.[6] Treating neuropathy is a difficult task for the physician and most of the conventional pain medications primarily mask symptoms.[7,8] and have significant side effects and addiction profiles. In the realm of physical medicine acupuncture, magnetic therapy, yoga have been found to provide benefit. One of the approaches which is currently of clinical interest includes low-frequency pulsed magnetic fields, which have analgesic, neurostimulatory, trophic, and vasoactive actions.[9] This article introduces and discusses the efficacy of low-frequency pulsed electromagnetic field which induces quasirectangular currents that can depolarize, repolarize, hyperpolarize neurons and can potentially modulate neuropathic pain and nerve impulse. It stimulates the cell power stations and enhances cell metabolism[10] resulting in higher mucosal content of RNA, DNA and improve the microcirculation due to an increased release of calcitonin gene related peptide-CGRP,[11,12] a bioactive messenger responsible for the formation of capillaries in wound area.

The aim of the present research was to study the effects of different frequencies (600 Hz, 800 Hz) of PEMF on motor nerve conduction parameters and pain control.

## Materials and Methods

This study was carried out in Guru Nanak Dev University and Government Medical college, Amritsar, India. The study was based on randomized purposive sampling technique in which all the aspects of age, sex, duration of diabetes, were matched except the treatment pattern in different groups. Thirty subjects (mean age 52 years) with type 2 diabetes (mean duration 12 years.) in stages N_1a_, N_1b_, N_2a_ of diabetic neuropathy as defined by Dyck and Thomas classification[13] were recruited for the present study. The battery of quantitative tests, which were included to confirm the diabetic neuropathy, was symptom questionnaire, clinical examination, quantitative sensory testing, nerve conduction study, and autonomic nervous system testing. Confirmed cases of diabetic neuropathy examined by the senior author (B.S. Bal) were taken as subject, based upon physical and neurological examination. Clinical records of each patients were reviewed, following which, nerve conduction studies was performed. Subjects with NCV 33-48 m/sec. with average 12-year-old diabetic history were recruited for the present study. Normal value of NCV in our lab setting in asymptomatic healthy volunteers in the same age group was reported to be 47–54 m/sec. Subjects included were having blood glucose under steady control for a period of three months prior to the study and were found to be refractory to various pain medications available for DPN. Subjects with only distal symmetric polyneuropathy were included. Subjects with peripheral vascular disease, history of major amputation, implantable medical devices, and other systemic disease that could potentially explain their symptoms were excluded. No new analgesic drug was allowed during the study, but individuals could remain on their current regimen of antidiabetic medication. The Institutional Medical Ethics Committee reviewed and approved the experimental protocol. The informed consent was obtained from all the subjects prior to study. 30 individuals were taken and randomly assigned to any one of the group 1, 2, 3 with 10 subjects in each group. The experimental groups 1, 2 were treated with low-frequency pulsed electromagnetic field of 600 and 800 Hz, respectively. The actions of PEMF were directed bilaterally on calf muscles of both lower limbs; each field was applied for 30 min. duration for 12 consecutive days. Group 3 served as control and received usual medical treatment of DPN. The efficacy of PEMF was assessed by VAS score and motor nerve conduction parameters. The baseline reading of pain on 11 point numeric pain rating scale (VAS; scale range: 0, no pain; 10, worse possible pain) and motor nerve conduction study (distal latency, nerve conduction velocity, amplitude) was done before and after treatment.

### Statistical analysis

Analysis was conducted with SPSS software. The results are expressed as mean± standard deviation. Related t-test and unrelated t-test were used for the intragroup and intergroup comparisons, respectively, to assess the statistical significance. A *‘P’* value of < 0.05 was considered to be significant.

## Results

**Figure 1:**
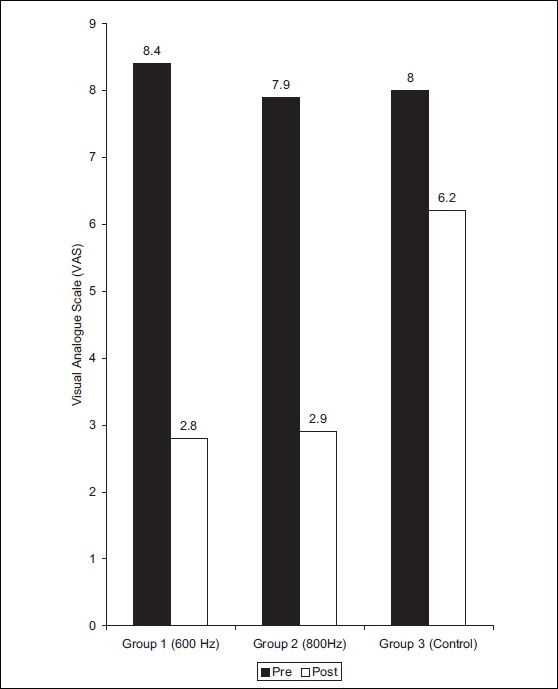
Distribution of mean values of VAS score on visual analogue scale in Group 1,2 and 3

The VAS score and nerve conduction study was repeated after treatment in experimental groups. The same readings were taken after 12 days for control group without any intervention. The VAS score decreased by 66.6, 63.25, and 22.5% in groups 1, 2, 3 respectively. There was a greater decrease in mean values for group 1 [Figure 1].

**Figure 2:**
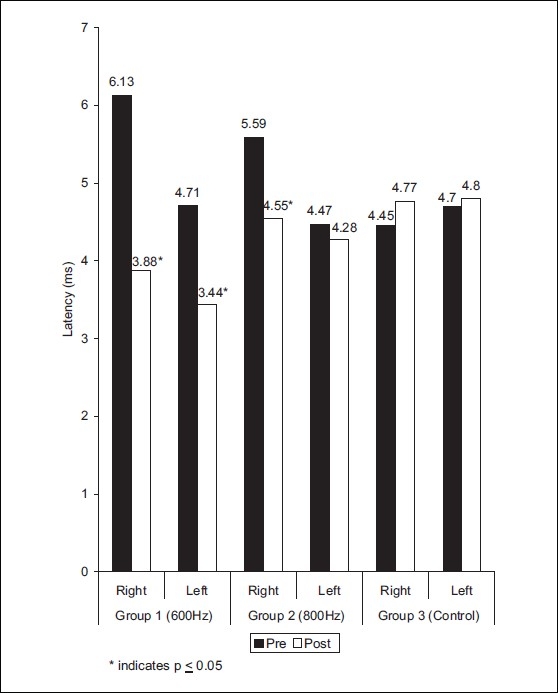
Distribution of mean values of latency in peroneal nerve at different frequencies in groups 1, 2, and 3

**Figure 3:**
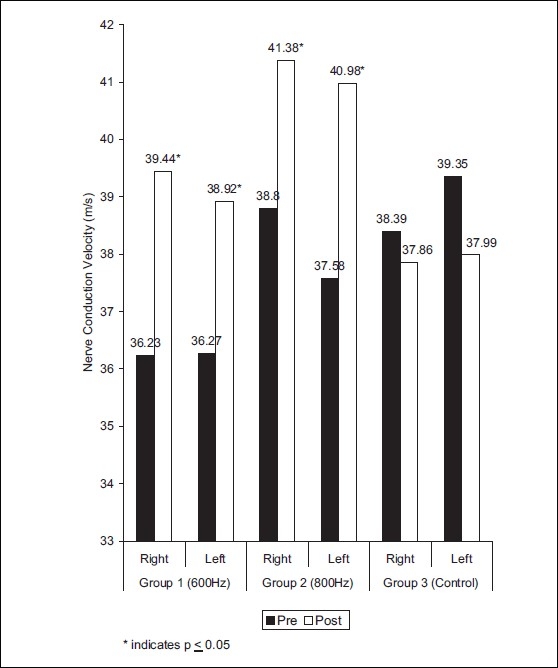
Distribution of mean values of nerve conduction velocity in peroneal nerve at different frequencies in groups 1, 2, and 3

The distal latency value in right peroneal nerve was 6.13 ± 3.18 and 3.88 ± 0.97 before and after treatment of group 1, respectively (*t* = 2.18) [Figure 2]. The mean values for NCV in right peroneal nerve were 36.23 ± 2.09 and 39.44 ± 2.83 before and after treatment, respectively *(t* = 2.89) [Figure 3]. In group 1, distal latency in left peroneal nerve was found to be 4.71 ± 0.99 and 3.44 ± 0.98 before and after treatment, respectively (*t* = 2.88) [Figure 2]. The mean values of NCV in left peroneal nerve were 36.27 ± 2.68, 38.92 ± 2.91 pre and post-treatment, respectively (*t* = 2.12) [Figure 3].

**Figure 4:**
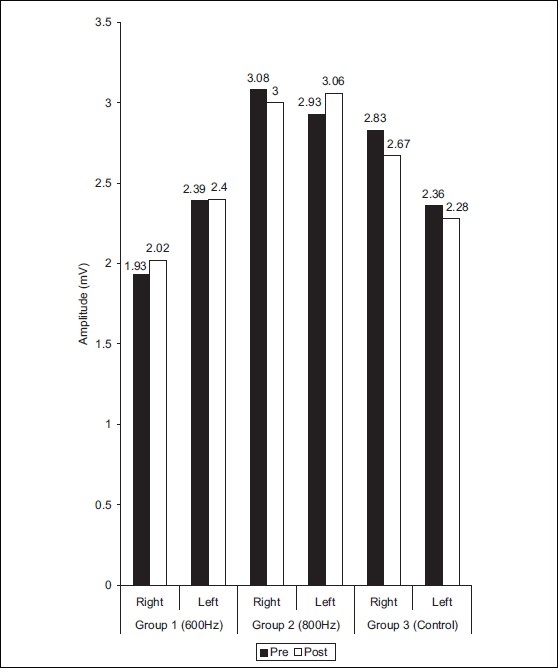
Distribution of mean values of amplitude in peroneal nerve at different frequencies in groups 1, 2, and 3

On left leg of group 2, the results for distal latency were 4.47± 0.96 and 4.28 ± 0.94 (*t* = 0.43) before and after treatment, respectively [Figure 2]. The mean values of NCV were 37.58 ± 6.15, 40.98 ± 6.63 (*t* = 2.56) in pre and post treatment conditions, respectively. On right leg of group 2, the results for distal latency, NCV were 5.59 ± 0.96, 4.55 ± 0.78 (t =2.6); 38.80 6.33, 41.38 ± 5.44 (*t* = 2.11) for pre and post-treatment condition, respectively [Figure 4]. The amplitude were found to be nonsignificant statistically in both the experimental groups [Figure 4].

In group 3, the distal latency in right peroneal nerve showed 4.45 ± 1.58, 4.77 ± 1.77 value before and after 12 days, respectively [Figure 2]. The NCV values for right peroneal nerve was 38.39 ± 6.41, 37.86 ± 6.47 before and after 12 days, respectively [Figure 3]. The left peroneal nerve showed distal latency 4.70 ± 1.11, 4.80 ± 1.34 before and after 12 days respectively [Figure 2]. The mean values of NCV were 39.35 ± 6.37, 37.99 ± 6.55 before and after 12 days [Figure 3]. The distal latency, NCV, amplitude were non significant (*P*<0.05) in control group.

## Discussion

In the present study, pulsed electromagnetic field therapy (600 and 800 Hz) was explored to study its effect on the state of segmental peripheral neuromotor apparatus and neuropathic pain experienced by patients. The primary aim of the study was to evaluate the effect of different frequencies of PEMF in diabetic polyneuropathy. In DPN, the pain may result due to various reasons such as increase in different signals from degenerating nociceptive afferent fibers, depolarization because of dysregulation of normal sodium,[14] calcium[15] and potassium[16] channel activities. It is well known that a biological system exposed to a physical stimulus (PEMF) is able to detect its presence and to modify its own biological activity depending on the characteristic of the applied stimulus such as mechanic, electric, or magnetic. In particular, static and time varying magnetic fields have been shown to alter animal and human behaviors such as pain perception.[17] The pain relief in experimental groups 1 and 2 could be attributed to the effect that magnetic fields affects pain perception by direct effects in form of neuron firing, calcium ion movement, endorphin levels, acupuncture action, and nerve regeneration.[18–20] A gating response with simultaneous stimulation of the Aδ fibers producing an inhibitory antinociceptive effect on C fibers which is compatible with Melzak–Wall Hypothesis.[21] The pain is most likely to arise from increased activity of injured small – diameter regenerating fibers,[15] which fire rapidly and at abnormally low thresholds.[22] The PEMF influence diabetic neurons and cell membrane of cutaneous nociceptors thereby inducing change in the cellular[23] and pericellular microenvironment.[24,25] The possible reason of pain reduction in group 3 is persistent controlled glycemic control. The strict glycemic control is a best measure to halt deterioration of DPN.[26]

The motor nerve conduction parameters (latency and NCV) showed significant improvement in group 1, 2 with better mean values in group 1. As demonstrated by Fagerberg's,[27,28] DPN is a result of diabetic angiopathy and found the correlation between neuropathic symptoms and duration of diabetes and histological abnormalities of the vasa nervorum.[29] The decrease in motor nerve conduction velocity (MCV) can be explained as a result of abnormality in the vasa nervorum. The experimental groups showed improvement in distal latency, NCV after treatment which can be attributed to indirect effect of PEMF, that is it augments angiogenesis by stimulating endothelial release of fibroblast growth factor beta – 2 (FGF – 2).[30] Smith[31] found that PEMF stimulate the arteriolar microvessel diameters in rat cremester muscle, which further support that it improves the microenvironment for the tissues leading to regeneration.[32,33] Other probable reason for the improvement may be that it stimulates neurotrophic factors that is known to play an important role in the development, maintenance, and survival of neuronal tissues.[3] Few studies suggested that endoneurial capillaries in peripheral nerves of the diabetes are thickened[29] and perineurial basement membrane are widened.[34] A permeability disorder at the blood nerve or blood perineurial barrier in diabetics could lead to endoneurial metabolic derangements, however possibly resulting in neuropathy. PEMF by targeting at increased circulation and anti inflammatory effects combined with the pain relief and restoration of normal nerve conduction lead to reversal of the damage that cause the peripheral neuropathy. Recently, it has been observed that PEMF modulates the neurite growth *in vitro* and nerve regeneration *in vivo,* which further explains the improvement obtained in results of group 1 and 2. None of the group showed significant changes in amplitude reading obtained in pre and post-treatment reading. The probable reason could be that amplitude of compound muscle action potential correlate with the number of nerve fibers recruited. As DPN is best classified as axonal neuropathy, in that predominant neuropathic feature is nerve fiber loss.[35] The effects of PEMF is to trigger a biologic response such as cell proliferation that represent the basic effect to explain some relevant results. It enhances nerve regeneration and accelerates recovery in experimentally divided and sutured peroneal nerve which can improve number of nerve fiber and thereby amplitude achieved in nerve conduction study. No difference in pre and post reading of amplitude could be attributed to short duration of treatment, which was inadequate to get the positive changes in amplitude. Comparative analysis showed non-significant differences in group 1, 2 after treatment. The mean difference was found to be more in group 1 (600 Hz) in both legs except NCV mean value which was higher in left leg of group 2 (800 Hz). The probable reason for this could be small sample size, short duration of study and individual variations.

In summary, it can be concluded that available data provide the evidence that PEMF treatment has the potential to modulate neuropathic pain and nerve impulse. It may be due to decrease in endoneural hypoxia, perineural edema, ischemia of peripheral nerves, and improved microcirculation that leads to positive changes after treatment sessions. The limitations of the study were small sample size, short term study, exclusion of patients other then distal symmetric polyneuropathy, and lack of follow-up.

## Conclusions

The present study provides convincing data regarding the effect of PEMF on neuropathic pain and nerve impulse. Considering its benefit and safety, low-frequency PEMF can be used as an adjunct in the management of diabetic neuropathy cases. Limitations of this study include small sample size, short duration of treatment, and nonavailability of follow-up data.
